# Diagnostic Innovations to Combat Antibiotic Resistance in Critical Care: Tools for Targeted Therapy and Stewardship

**DOI:** 10.3390/diagnostics15172244

**Published:** 2025-09-05

**Authors:** Ahmed D. Alatawi, Helal F. Hetta, Mostafa A. Sayed Ali, Yasmin N. Ramadan, Amirah B. Alaqyli, Wareef K. Alansari, Nada H. Aldhaheri, Talidah A. Bin Selim, Shahad A. Merdad, Maram O. Alharbi, Wejdan Alhumaidi Hmdan Alatawi, Abdelazeem M. Algammal

**Affiliations:** 1 Department of Clinical Pharmacy, College of Pharmacy, Jouf University, Sakaka 72388, Saudi Arabia; adalatawi@ju.edu.sa; 2 Division of Microbiology, Immunology and Biotechnology, Department of Natural Products and Alternative Medicine, Faculty of Pharmacy, University of Tabuk, Tabuk 71491, Saudi Arabia; 3 Department of Pharmacy Practice, Faculty of Pharmacy, University of Tabuk, Tabuk 71491, Saudi Arabia; ma-ali@ut.edu.sa; 4 Department of Microbiology and Immunology, Faculty of Pharmacy, Assiut University, Assiut 71515, Egypt; yasmine_mohamed@pharm.aun.edu.eg; 5 College of Applied Medical Sciences, University of Tabuk, Tabuk 47315, Saudi Arabia; dr.ameerah2001@gmail.com; 6 Medical Laboratory Technology, College of Applied Medical Sciences, University of Taibah, Medina 42367, Saudi Arabia; wareefalansari2@gmail.com (W.K.A.); nadaldhaheri@gmail.com (N.H.A.); 7 Laboratory Medicine, College of Applied Medical Sciences, Umm Al-Qura University, Makkah 24321, Saudi Arabia; talidah.binselim@gmail.com (T.A.B.S.); s441009129@uqu.edu.sa (M.O.A.); 8 Medical Laboratory Technology, College of Applied Medical Sciences, University of Northern Border, Arar 73213, Saudi Arabia; drwejdanalhumaidi@gmail.com; 9 Department of Bacteriology, Immunology and Mycology, Faculty of Veterinary Medicine, Suez Canal University, Ismailia 41522, Egypt

**Keywords:** antimicrobial resistance, intensive care unit (ICU), rapid diagnostics, point-of-care (POC) testing, diagnostic stewardship

## Abstract

Antibiotic resistance is a growing global health threat, with critical care settings representing one of the most vulnerable arenas due to the high burden of infection and frequent empirical antibiotic use. Rapid and precise diagnosis of infectious pathogens is crucial for initiating appropriate therapy, minimizing unnecessary antimicrobial exposure, and supporting effective stewardship programs. This review explores how innovative diagnostic technologies are reshaping infection management and antimicrobial stewardship in critical care. We examine the clinical utility of molecular assays, multiplex PCR, MALDI-TOF mass spectrometry, metagenomic sequencing, point-of-care (POC) diagnostics, and emerging tools like biosensors and AI-powered predictive models. These platforms enable earlier pathogen identification and resistance profiling, facilitating timely and targeted therapy while minimizing unnecessary broad-spectrum antibiotic use. By integrating diagnostics into stewardship frameworks, clinicians can optimize antimicrobial regimens, improve patient outcomes, and reduce resistance selection pressure. Despite their promise, adoption is challenged by cost, infrastructure, interpretation complexity, and inequitable access, particularly in low-resource settings. Future perspectives emphasize the need for scalable, AI-enhanced, and globally accessible diagnostic solutions. In bridging innovation with clinical application, diagnostic advancements can serve as pivotal tools in the global effort to curb antimicrobial resistance in critical care environments.

## 1. Introduction

Antimicrobial resistance (AMR) is recognized as a major global health challenge of the 21st century, accounting for an estimated 4.95 million deaths annually, with 1.27 million directly attributable to drug-resistant infections according to the most recent Global Burden of Disease analysis [1,2]. The problem is particularly acute in intensive care units (ICUs), where critically ill patients are at increased risk of multidrug-resistant organism (MDRO) acquisition due to invasive interventions, prolonged hospitalization, and frequent use of broad-spectrum empirical antibiotics [3,4,5,6,7].

Accurate and timely diagnosis of infectious etiologies in intensive care units (ICUs) is often limited by the constraints of conventional microbiological techniques. Although culture-based methods remain the diagnostic reference standard, they are hindered by prolonged turnaround times, reduced sensitivity after prior antibiotic exposure, and difficulties in detecting polymicrobial or fastidious organisms [8,9,10,11]. These limitations frequently result in the empiric administration of broad-spectrum antibiotics, which may accelerate resistance development, increase the length of ICU stay, and raise healthcare costs [11].

To address these challenges, significant progress has been made in the development of advanced diagnostic modalities. Molecular assays, syndromic panels, mass spectrometry, and metagenomic approaches provide rapid pathogen identification and resistance profiling, enabling the timely initiation or de-escalation of antibiotic therapy [12,13,14].

In this context, diagnostics emerge not as passive tools but as active enablers of antimicrobial stewardship. Their integration into stewardship frameworks is increasingly recognized as a critical strategy to optimize antibiotic use, prevent unnecessary exposure, and mitigate the risk of resistance [8]. However, the successful translation of diagnostic advancements into routine critical care practice remains challenged by barriers related to cost, infrastructure, and interpretative complexity.

This review aims to provide a comprehensive overview of diagnostic innovations currently shaping the landscape of infection management in ICUs. By examining the utility, limitations, and stewardship potential of emerging tools, we highlight the indispensable role of diagnostics in combating antibiotic resistance and promoting precision-guided therapy in critical care.

This review was conducted as a narrative synthesis with elements of a semi-systematic approach to ensure comprehensive coverage of diagnostic innovations relevant to antimicrobial resistance management in critical care. The literature was identified through searches of the PubMed, Scopus, and Web of Science databases for articles published between January 2010 and May 2025, using combinations of keywords such as “antimicrobial resistance”, “rapid diagnostics”, “critical care”, “ICU”, “point-of-care testing”, “molecular diagnostics”, “MALDI-TOF”, “metagenomics”, and “diagnostic stewardship”. Additional sources were identified by screening reference lists of relevant articles. Inclusion criteria encompassed peer-reviewed studies, systematic reviews, meta-analyses, and key expert consensus statements focusing on human clinical applications or stewardship frameworks in ICU settings. Exclusion criteria included studies without relevance to critical care, those focused solely on veterinary or environmental contexts, and non-English-language publications.

## 2. Challenge of Antibiotic Resistance in Critical Care

The ICU is a high-risk environment for antimicrobial resistance due to severe patient illness, invasive procedures, and extensive antibiotic use [15,16,17,18,19]. This significantly compromises infection management.

Critically ill patients are uniquely susceptible to infections due to multiple risk factors, including immunosuppression, invasive devices (such as central venous catheters, mechanical ventilation, and urinary catheters), prolonged hospital stays, and frequent exposure to broad-spectrum antibiotics [20,21,22,23,24,25]. These factors elevate the risk of healthcare-associated infections (HAIs) and promote colonization by MDROs such as carbapenem-resistant *K. pneumoniae*, methicillin-resistant *Staphylococcus aureus* (MRSA), and multidrug-resistant *A. baumannii* [5,26,27]. According to the GBD 2021 [28,29] study, six pathogens—*S. aureus*, *A. baumannii*, *E. coli*, *K. pneumoniae*, *S. pneumoniae*, and *P. aeruginosa*—were responsible for an attributable AMR burden of at least 100,000 deaths in 2021, with *S. aureus* and *A. baumannii* among the top contributors. These pathogens are particularly prevalent in ICU settings, where their high levels of resistance to last-resort antibiotics like carbapenems and colistin exacerbate the challenges in treating severe infections. These data underscore not only the pervasive threat posed by these specific pathogens but also the growing burden of antimicrobial resistance in critical care environments, where timely and effective treatment is often a matter of life or death.

The urgency and severity of infections in the ICU often necessitate the empirical use of antibiotics before a definitive microbiological diagnosis can be made. While this approach is frequently life-saving, it also contributes to the overuse and misuse of antibiotics, especially when treatment is not promptly tailored based on culture results or when the infection is non-bacterial in origin [4]. A study by Vincent et al. reported that more than 70% of ICU patients receive antibiotics, yet only about half of these treatments are confirmed to target a documented infection [30]. This empirical overuse exacerbates selective pressure, accelerating the emergence and spread of resistance.

Moreover, conventional diagnostic methods in critical care are frequently insufficient to meet the time-sensitive demands of infection management. Culture-based diagnostics may take 48–72 h or longer to yield actionable results, during which time patients often receive prolonged and unnecessary broad-spectrum therapy [31]. Inadequate or delayed therapy in the presence of resistant pathogens is independently associated with higher mortality, particularly in bloodstream infections and ventilator-associated pneumonias [32,33].

The clinical consequences of AMR in critical care settings are profound. Infections caused by resistant organisms are associated with increased morbidity, length of ICU stay, mechanical ventilation duration, and healthcare costs, as well as significantly higher mortality rates compared to susceptible infections [34]. Furthermore, the spread of resistance within ICUs has broader epidemiological implications, as these units often serve as epicenters for transmission both within hospitals and across healthcare networks [35].

Addressing this multifaceted challenge requires a paradigm shift toward early, accurate, and actionable diagnostics that inform rational antibiotic use. Rapid identification of pathogens and their resistance profiles is essential for narrowing therapy, minimizing unnecessary exposure, and ultimately curbing the selection of resistant strains. In this context, diagnostic innovations contribute directly to stewardship interventions [36].

## 3. Diagnostic Innovations as Tools for Targeted Therapy

Early, accurate, and clinically relevant diagnostics are essential to guide antimicrobial therapy in the ICU. Diagnostic platforms can be broadly categorized based on their primary function: (1) pathogen identification, (2) resistance detection, and (3) host response assessment. Organizing tools in this way mirrors the clinical decision-making process, moving from confirming the infectious agent to understanding its resistance profile and, finally, evaluating the host’s immune response. The readiness of these technologies for ICU implementation varies, ranging from fully established tools to experimental platforms still in validation. A summary of their clinical maturity and current adoption status is provided in Table 1. Moreover, a comparative overview of these technologies, including performance characteristics, cost considerations, and clinical impact, is summarized in Table 2.

### 3.1. Pathogen Identification

#### 3.1.1. Molecular and Genotypic Tools

In critical care settings, timely and accurate pathogen identification is essential to guide effective antimicrobial therapy. Traditional culture-based diagnostics, while foundational, are often hindered by long turnaround times. The introduction of molecular and genotypic tools has revolutionized diagnostics by enabling the rapid detection of pathogens and key resistance genes, often within just a few hours.

Two of the most widely adopted platforms in intensive care settings are the BioFire^®^ FilmArray^®^ Blood Culture Identification (BCID) Panel and the Cepheid GeneXpert^®^ system. These systems allow for direct-from-specimen testing, minimizing delays associated with subculturing or susceptibility testing [37,38].

#### 3.1.2. MALDI-TOF Mass Spectrometry (MS)

Matrix-Assisted Laser Desorption Ionization Time-of-Flight Mass Spectrometry (MALDI-TOF MS) has revolutionized clinical microbiology by offering rapid, reliable, and cost-effective identification of microbial species. Its integration into intensive care diagnostics has significantly shortened the time required to identify pathogens—one of the major bottlenecks in managing infections in critically ill patients [39].

MALDI-TOF MS provides rapid microbial identification by analyzing unique protein fingerprints from samples, with results available in minutes. The process begins with the application of a microbial colony or direct sample onto a metal target plate, followed by overlay with a matrix solution. Once dried, the sample is exposed to a laser, which causes ionization and desorption of the proteins. These ionized particles are then accelerated through a vacuum tube, where their time of flight (TOF) is measured. Because each microorganism has a characteristic protein mass spectrum, the result is compared against an extensive reference database to determine the species with high accuracy—often in under 15 min from colony growth [39,40,41].

In the ICU setting, MALDI-TOF MS is particularly valuable for bloodstream infections, pneumonia, urinary tract infections, and intra-abdominal infections, where rapid decisions regarding antimicrobial therapy are crucial. Recent advancements now enable direct identification from positive blood cultures, bypassing the need for overnight subculturing and thereby reducing the turnaround time by up to 24 h [42,43,44].

While the core application is species identification, emerging workflows and complementary assays now allow the detection of antimicrobial resistance mechanisms, such as carbapenemase or extended-spectrum β-lactamase (ESBL) activity, by observing specific mass shifts or hydrolysis patterns in the presence of antibiotics [43].

The clinical impact of MALDI-TOF MS is evident when it is embedded in a coordinated antimicrobial stewardship program. It has been shown to reduce unnecessary antibiotic use, improve patient outcomes, and shorten hospital stays in critically ill patients [45,46,47]. However, one limitation is its inability to directly detect resistance genes or predict susceptibility profiles without adjunctive molecular or phenotypic testing. Nonetheless, the high throughput, reproducibility, and low per-sample cost make MALDI-TOF MS an indispensable tool in modern microbiology laboratories, particularly in the high-stakes environment of critical care [47].

Despite these advantages, MALDI-TOF MS has notable practical limitations in the ICU context. Optimal accuracy generally requires a pure culture, as mixed-species samples or closely related organisms can produce overlapping spectra that complicate interpretation. Furthermore, differences between commercial systems—such as those from Bruker and bioMérieux—mean that spectral databases are not cross-compatible. As a result, laboratories switching between platforms cannot directly transfer their accumulated spectral libraries and must rebuild or revalidate them, which is resource- and time-intensive. These factors should be considered when planning MALDI-TOF implementation or transitioning between systems [41,48,49].

#### 3.1.3. Point-of-Care and Bedside Tools for Identification

**Figure 1:**
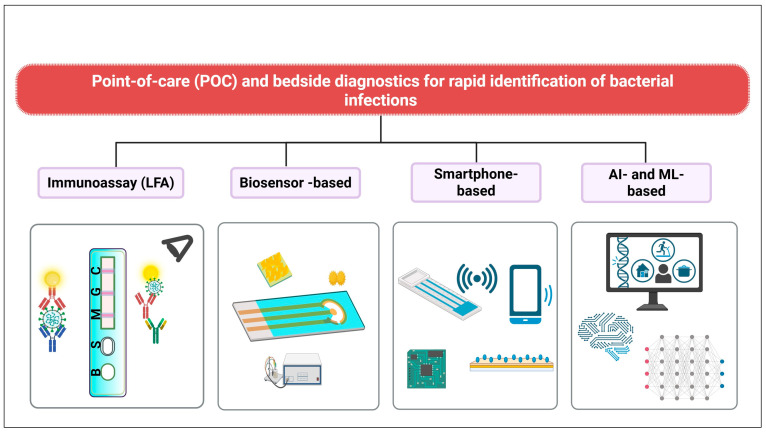
Overview of diagnostic modalities for combating antimicrobial resistance in critical care. The diagram groups technologies according to their primary detection principles (e.g., molecular, immunological, biosensor-based) and data analysis approaches (conventional algorithms vs. AI-enhanced interpretation). Device location (e.g., smartphone-integrated vs. standalone laboratory systems) is indicated because portability and bedside integration can directly impact turnaround time, workflow compatibility, and applicability in ICU environments. Created with BioRender.com.

In critical care settings, timely identification of infections is vital to initiating appropriate antimicrobial therapy and reducing unnecessary antibiotic exposure. Point-of-care (POC) and bedside diagnostic innovations have emerged as effective tools in this context, enabling clinicians to rapidly assess infectious conditions without relying on centralized laboratory infrastructure (Figure 1). These technologies, tailored for speed, portability, and clinical integration, complement antimicrobial stewardship strategies by supporting early diagnosis, targeted treatment, and real-time monitoring [50,51,52].

#### 3.1.4. Lateral Flow Assays (LFAs)

**Figure 2:**
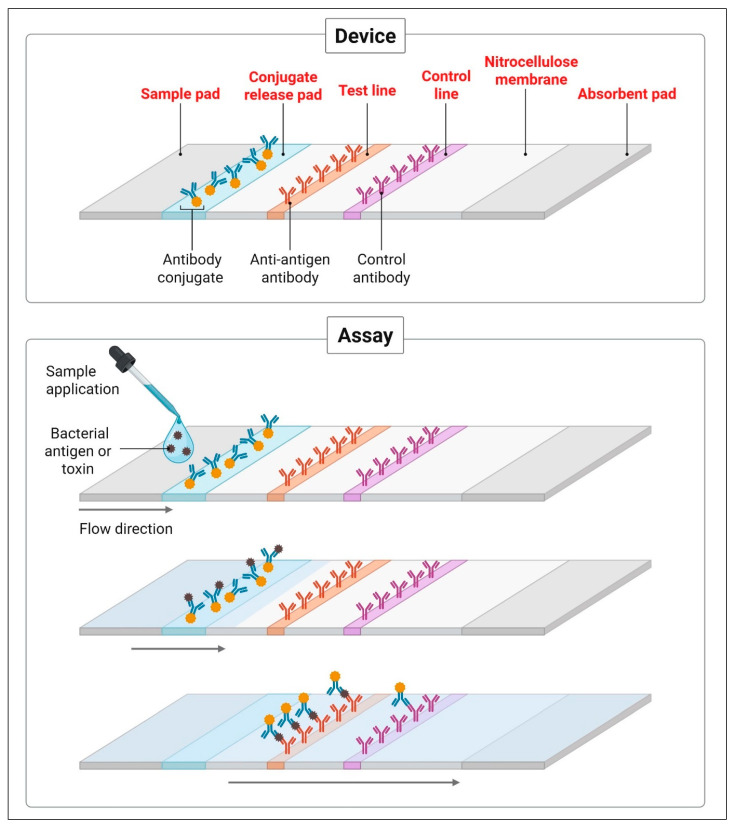
Schematic representation of a lateral flow immunoassay (LFA) for bacterial antigen or toxin detection. The device consists of a sample pad, conjugate release pad, test and control lines on a nitrocellulose membrane, and an absorbent pad. The assay mechanism involves sample application, antigen–antibody interaction, and visual signal development at the test and control lines, enabling rapid and on-site pathogen identification. Created with BioRender.com.

Lateral flow assays (LFAs) represent one of the most widely adopted POC diagnostic platforms due to their simplicity, rapidity, and portability. These immunochromatographic assays function by detecting specific microbial antigens or toxins in a patient sample—such as stool, urine, nasopharyngeal swabs, or blood—through antigen–antibody interactions on a nitrocellulose membrane strip. Results are typically available within 10–30 min and can be interpreted visually or digitally via integrated readers [53,54] (Figure 2).

Lateral flow assays (LFAs), established in ICU workflows for specific bacterial and viral targets, are widely used for *C. difficile*, *Legionella*, *S. pneumoniae*, influenza, and SARS-CoV-2. Multiplex LFAs with enhanced sensitivity are emerging and undergoing clinical validation [13,50,53,55,56].

Importantly, Bouzid et al. [13] emphasized that in emergency departments, LFAs substantially contribute to earlier clinical decision-making and patient stratification. Their analysis revealed that LFAs shortened diagnostic delays, reduced unnecessary antibiotic use, and enhanced pathogen-specific diagnosis in acute care settings. This aligns with antimicrobial stewardship goals, particularly in the ICU where every hour of delayed appropriate therapy is associated with increased mortality in sepsis and severe infections.

Despite their rapid turnaround and ease of use, LFAs generally exhibit lower sensitivity compared to molecular diagnostics, particularly when pathogen loads are low. However, when incorporated into clinical workflows—especially in high-prevalence scenarios or as part of an initial screening step—they can significantly enhance diagnostic efficiency and reduce empirical antibiotic prescribing [57].

Next-generation LFAs now incorporate multiplex and multianalytical platforms—using fluorescence, surface-enhanced Raman scattering (SERS), or electrochemical readouts—to enhance sensitivity and enable the simultaneous detection of multiple pathogens or biomarkers. Such capabilities are especially useful for complex ICU syndromes like ventilator-associated pneumonia or bloodstream infections. Digital readers and mobile-linked biosensor systems further extend their utility in bedside and resource-limited environments [58]. Despite these advances, LFAs remain constrained by their generally lower sensitivity compared to nucleic acid-based assays, particularly in cases of low pathogen burden. Most multiplex LFA platforms are still in early validation and may not yet have regulatory approval for ICU use. Additionally, single-use cartridges can increase per-test costs in high-volume settings, and interpreting faint visual bands without digital readers may introduce user-dependent variability [59].

#### 3.1.5. Biosensors and Lab-on-a-Chip Platforms for Pathogen Identification

Biosensors and lab-on-a-chip (LOC) platforms represent a transformative impact in infectious disease diagnostics—particularly valuable in the intensive care setting, where rapid, accurate, and bedside-compatible tools are essential. These systems combine microfluidics, biorecognition elements, and transducers to detect biological markers of infection with high sensitivity and short turnaround times, often using just a few microliters of sample [60,61].

A biosensor is a compact device designed to detect biological molecules quickly and accurately. It typically has three key components: a biological recognition element that binds specifically to the target (such as a bacterial antigen, a resistance gene, or a metabolic byproduct); a transducer that converts this biological interaction into a measurable signal (like light, electric current, or vibrations); and a signal processor that interprets and displays the result. This process allows biosensors to detect pathogens or resistance mechanisms directly from clinical samples without the need for complex lab equipment [61].

From practical view, most biosensors remain experimental, with promising prototypes in pilot testing; few are in regular ICU use. For example, immunosensors can detect bacterial surface antigens or toxins from organisms like *E. coli*, *S. aureus*, or *P. aeruginosa* [62]. Other biosensors measure volatile organic compounds (VOCs) or metabolites that are released by bacteria during infection [63,64].

LOC platforms enhance these capabilities by integrating sample preparation, amplification, and detection in a single device, allowing rapid, multiplexed analysis of specimens such as blood, urine, or respiratory secretions. Their speed and low sample requirements make them particularly suited for ICU environments where rapid, precise decisions are needed.

Despite their potential, most biosensors and LOC platforms remain in early-stage clinical validation and lack broad regulatory approval for ICU use. Production and implementation costs can be high, and some designs require specialized handling or maintenance, limiting their applicability in resource-constrained settings. Additionally, variations in sample matrix composition may affect analytical performance, and large-scale, multi-center studies are still needed to confirm reliability in diverse patient populations [65].

#### 3.1.6. Smartphone-Based Diagnostics for Pathogen Identification

The combination of mobile technology and POC diagnostics has led to the development of smartphone-based diagnostic tools. This approach is experimental, primarily in research settings or limited pilot studies, and not yet part of routine ICU protocols. These platforms are becoming valuable in managing infectious diseases, especially in busy or low-resource settings like ICUs. They typically connect smartphones with devices such as biosensors, LFAs, microfluidic chips, or fluorescence readers. This setup allows fast testing and immediate analysis at the bedside. Smartphones use their built-in cameras, wireless features, and apps to read test results, measure color changes, and even help identify bacteria. This improves the accuracy of diagnosis and reduces human error.

Several recent studies highlight the diagnostic potential of smartphone-enabled platforms in bacterial detection and antimicrobial stewardship. For instance, Lin et al. developed a smartphone-assisted and handheld fluorometer platform for the rapid detection of *E. coli* in urinary tract infections (UTIs), utilizing a novel mechanism of specific proteolytic cleavage followed by cascade amplification [66]. This method offered high sensitivity and specificity within minutes, making it highly suitable for bedside decision-making in critical care environments. Similarly, Yin et al. developed a smartphone-based fluorescent sensor that rapidly identified multiple bacterial pathogens by combining magnetic separation with fluorescent signal amplification. This approach enabled the multiplexed and highly sensitive detection of pathogens such as *E. coli*, *S. aureus*, and *P. aeruginosa*, achieving a detection limit of 10^2^ CFU/mL within 40 min [67]. Moreover, Zhou et al. introduced a smartphone-based polydiacetylene colorimetric sensor capable of detecting bacterial infections through visible color change [68]. Their device demonstrated robust accuracy, minimal equipment requirements, and strong potential for use in bedside diagnostics and home-based monitoring. Complementing these innovations, Pawar et al. provided a comprehensive overview of smartphone-based biosensing platforms targeting a broad range of human pathogens [69]. Their analysis highlights how smartphones, when integrated with lateral flow assays, optical sensors, or microfluidic chips, can enhance diagnostic accuracy, support early detection, and improve therapeutic outcomes—especially in low-resource or remote areas.

### 3.2. Resistance Detection

Once a pathogen is identified, determining its resistance profile is essential for guiding targeted therapy and avoiding unnecessary broad-spectrum antibiotic use. Resistance detection technologies can identify specific genetic determinants or reveal phenotypic resistance mechanisms, providing actionable information for antimicrobial stewardship in the ICU.

#### 3.2.1. Molecular Detection of Resistance Genes

Molecular assays enable the rapid detection of key resistance markers directly from clinical samples or positive cultures, often delivering results in under two hours.

The BioFire^®^ BCID panel was established and widely deployed in ICUs worldwide for bloodstream infection management. For example, it identifies more than 20 common bloodstream pathogens along with critical resistance genes—such as *mecA*, *vanA/B*, and *blaKPC*—within approximately one hour from positive blood culture bottles. Its multiplex PCR approach supports syndromic testing for presentations like sepsis, significantly improving the time to targeted therapy [14,70].

Similarly, the Cepheid GeneXpert^®^ system was established, with common use in ICU screening protocols for MRSA, carbapenemase-producing organisms, and infection control. For example, the Xpert^®^ Carba-R assay rapidly identifies carbapenemase genes (*KPC*, *NDM*, *OXA-48*, *VIM*, *IMP*) from rectal swabs or other clinical materials—critical for infection control and therapy selection in ICUs [71,72]. The Xpert^®^ MRSA/SA tests are also widely used in ICU screening, enabling timely de-escalation or focused therapy [73].

When integrated into stewardship programs, these platforms have been shown to shorten the time to effective treatment, reduce broad-spectrum antibiotic use, decrease ICU length of stay, and potentially improve survival in bloodstream infections and sepsis [73]. Although their upfront costs are higher than conventional testing, the downstream benefits—through reduced diagnostic uncertainty, optimized antibiotic targeting, and better patient outcomes—justify their adoption in critical care [74].

#### 3.2.2. Phenotypic Resistance Testing via Biosensors and LOC Platforms

Phenotypic platforms provide functional evidence of resistance, complementing molecular assays by detecting the actual expression of resistance mechanisms.

Lab-on-a-chip (LOC) systems integrate sample preparation, processing, and detection into a single microfluidic device, significantly reducing the turnaround times for antimicrobial susceptibility testing (AST) [75,76,77]. LOC AST devices are emerging, with several undergoing early clinical pilot testing, but they are not yet standard in most ICUs. In a systematic review, Ardila et al. reported that LOC platforms achieved high sensitivity and specificity for detecting *S. aureus* susceptibility profiles, often with minimal sample volumes and reduced technical requirements—features especially valuable in resource-limited ICUs [78].

In a systematic review, Ardila et al. reported that LOC platforms achieved high sensitivity and specificity for detecting *S. aureus* susceptibility profiles, often with minimal sample volumes and reduced technical requirements—features especially valuable in resource-limited ICUs [79]. LOC devices integrating isothermal amplification methods such as LAMP with real-time optical detection have successfully identified multidrug-resistant organisms like MRSA and *K. pneumoniae* directly from clinical specimens [80]. These systems combine genetic and functional data, making them highly suitable for point-of-care resistance testing.

Nucleic acid-based biosensors further expand this capability by detecting key genetic markers, such as *blaKPC* or *mecA*, directly from primary samples [12]. Notably, these nucleic acid-based biosensors are mostly experimental, with limited clinical integration.

#### 3.2.3. Rapid Antimicrobial Susceptibility Testing (rAST)

Rapid Antimicrobial Susceptibility Testing (rAST) refers to phenotypic methods capable of providing actionable susceptibility results in as little as 2–8 h, substantially shortening the time to targeted therapy compared with conventional broth microdilution or disk diffusion methods, which often require 18–48 h after pathogen identification. By delivering early susceptibility profiles—often directly from positive blood cultures—rAST platforms help bridge the gap between rapid pathogen identification and optimal antimicrobial selection, a critical step in ICU stewardship programs [81,82].

Several commercially available rAST platforms have been developed with intensive care applications in mind, and some have already obtained regulatory clearance (FDA or CE-IVD). The Alfred 60/AST (Alifax) uses light-scattering technology to monitor bacterial growth in the presence of antibiotics, generating results in approximately 5–6 h with high categorical agreement rates for both Gram-negative (~95%) and Gram-positive (~89%) organisms [83]. FASTinov (FASTinov SA) applies ultrarapid flow cytometry to detect early physiological changes in bacteria exposed to antibiotics, producing reliable susceptibility profiles in under 2 h directly from positive blood cultures [84,85]. This system has demonstrated high accuracy across multiple sites and the ability to detect specific resistance mechanisms such as plasmid-mediated AmpC β-lactamases [86]. The LifeScale system (Affinity Biosensors) employs microfluidic sensors for real-time growth monitoring, enabling MIC determination in about 6 h and holding both FDA clearance and CE-IVD approval for several assays [87,88]. VITEK^®^ REVEAL (bioMérieux) integrates advanced imaging and growth detection algorithms to deliver results in 5–7 h, with design features that support seamless integration into routine laboratory workflows [89,90]. Other platforms include the ASTar system, a fully automated CE-IVD system capable of providing MIC results in roughly 6 h with minimal hands-on time [91]. The Quantamatrix QMAC-dRAST system has also been prospectively evaluated for direct testing from positive blood cultures and shown good agreement with reference methods while providing results within approximately 6 h [38,92,93]. Newer microfluidic solutions such as the Selux NGP system have been introduced to the market as additional rapid phenotypic options [94]; pilot and validation reports continue to expand the evidence base. Abstract and conference data further support that accurate carbapenem susceptibility testing can be achieved within 5–6 h on some rapid platforms [95].

Clinical studies indicate that integrating rAST into ICU workflows can shorten the time to appropriate, targeted therapy—often by 12–24 h—thereby supporting the earlier de-escalation of broad-spectrum agents, faster escalation when necessary, and potential improvements in clinical outcomes. However, several practical and regulatory considerations present barriers to implementation for immediate universal uptake. Platforms differ in analytical approach, organism–antibiotic coverage, and categorical agreement with reference methods for specific organism–antibiotic combinations; some panels remain limited in scope. Implementation requires substantial capital investment, ongoing consumable costs, local validation for each organism–drug pair, and adjustments to laboratory workflows and reporting pathways. Moreover, evolving regulatory landscapes for rapid AST and laboratory-developed tests are likely to affect clinical practice and adoption [96], and clinical stewardship impact should ideally be confirmed in larger pragmatic trials. In summary, while rAST technologies represent an important advance toward timely, phenotype-based guidance for ICU antimicrobial decisions, their real-world value depends on the careful selection of platforms, local validation, and integration into stewardship protocols.

#### 3.2.4. Smartphone-Based Diagnostics for Resistance Detection and Surveillance

Smartphone-integrated platforms extend resistance detection capabilities beyond traditional laboratory settings. Combining portable biosensors with AI algorithms, they can analyze diagnostic readouts in real time, increasing precision and reducing operator variability.

For example, Wen et al. developed a smartphone-based surface plasmon resonance (SPR) sensing system that, aided by AI-driven image analysis, rapidly quantified bacterial concentrations without complex instrumentation [97]. When linked to cloud-based platforms and electronic health records (EHRs), these tools can provide immediate access to patient-specific resistance data, generate automated clinical alerts, and support dynamic therapy adjustments.

At a broader scale, aggregated data from smartphone diagnostics contribute to public health surveillance, mapping local and regional AMR trends and enabling earlier outbreak detection. This dual role—in patient-level guidance and population-level monitoring—makes them a promising adjunct to both ICU stewardship programs and global AMR containment strategies.

### 3.3. Host Response-Based Diagnostics

Host response-based diagnostics evaluate the patient’s immune and physiological reaction to infection, providing insights that go beyond pathogen identification. By assessing biomarkers and integrating clinical data, these tools can help differentiate infectious from non-infectious causes, determine infection severity, and guide therapy duration—key components of precision antimicrobial stewardship in the ICU.

#### 3.3.1. Biomarker Detection

Advanced biosensors are increasingly designed to detect host-derived biomarkers, such as interleukins and procalcitonin (PCT), which can inform early diagnosis, assess infection severity, and support decisions on initiating or discontinuing antibiotic therapy [98,99]. PCT-guided stewardship strategies, for example, have been shown to safely reduce antibiotic exposure in sepsis and lower respiratory tract infections, while serial interleukin measurements can help monitor treatment response. Integration of these biomarker assays into rapid, point-of-care formats enhances their clinical utility, enabling bedside decision-making within minutes.

#### 3.3.2. AI-Powered Predictive Tools in Antimicrobial Resistance Management

**Figure 3:**
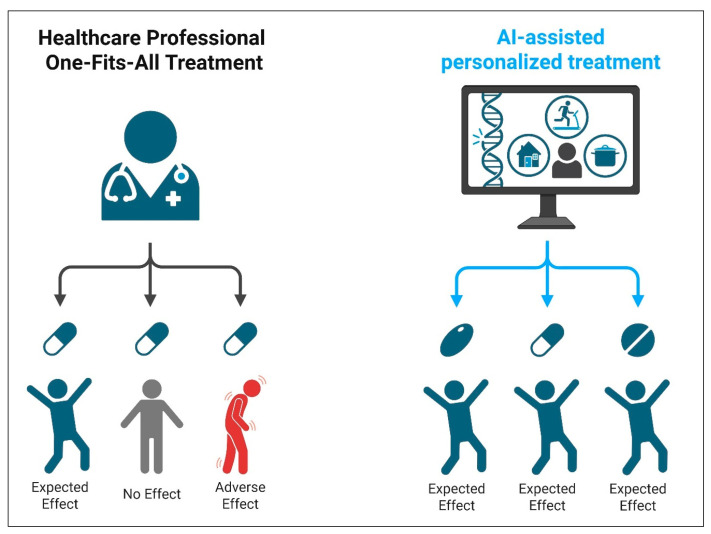
Schematic representation of AI-assisted diagnostic workflows in antimicrobial resistance management. AI models, such as those based on machine learning, analyze diagnostic and clinical data to generate predictions with measurable accuracy (sensitivity, specificity, predictive values). These predictions are designed to complement—not replace—clinician judgment. Final therapeutic decisions should be based on a synthesis of diagnostic data, patient presentation, and professional expertise. Created with BioRender.com.

Artificial intelligence (AI) and machine learning (ML) assist antimicrobial resistance (AMR) management by analyzing clinical data to predict resistance patterns and optimize therapy. These systems can analyze large, heterogeneous datasets—such as patient demographics, prior antibiotic exposure, comorbidities, local resistance patterns, microbial genomic profiles, and EHR data—to predict the likelihood of resistance and guide empirical therapy choices. However, their performance is not “infallible” and depends on the quality, representativeness, and completeness of the underlying data. Machine learning models have quantifiable sensitivity and specificity, and their predictive outputs should be interpreted as supportive information rather than definitive instructions. Final decisions on ordering diagnostics or initiating treatment remain the responsibility of the treating physician, integrating AI insights with the patient’s clinical context [100,101,102] (Figure 3). This targeted approach supports both improved patient outcomes and reduced misuse of broad-spectrum agents.

Various AI- and ML-based models have demonstrated clinical potential. Decision tree models (e.g., random forests) and deep learning networks have accurately predicted resistance to carbapenems and other critical antibiotics in ICU bloodstream infections [103,104,105,106]. Logistic regression and support vector machine models have also been trained on EHR and microbiology datasets to guide antibiotic selection, incorporating variables such as hospitalization length, invasive device use, and prior treatment history [107].

Beyond prediction, AI-driven decision support systems (DSSs) are increasingly embedded in hospital IT frameworks. These systems integrate predictive analytics with live clinical workflows, offering automated, antibiogram-informed therapy recommendations. They can also function as AI-powered surveillance tools, continuously monitoring AMR trends within hospital units and across geographic regions to enable early outbreak detection and rapid containment [108,109,110].

Despite these advantages, challenges persist. Data standardization across institutions, algorithm interpretability, and the risk of automation bias remain barriers to clinician trust and widespread adoption [111,112]. Ethical and regulatory concerns, particularly regarding patient data privacy under frameworks such as GDPR and HIPAA, must be addressed when designing and deploying AI tools [113,114,115]. Emerging hybrid approaches that combine clinical rules with AI predictions may improve acceptance and reliability [116,117]. Future developments may also enable the prediction of horizontal gene transfer events or real-time detection of clonal outbreaks through hospital-wide genomic surveillance.

Continued collaboration among data scientists, microbiologists, infectious disease clinicians, and health IT teams will be essential to enhance the accuracy, scalability, and interpretability of AI for AMR surveillance and intervention, ultimately supporting safer and more effective antimicrobial stewardship in the ICU [108,118].

Despite promising results in pilot and early clinical studies, most AI-based decision support platforms remain under validation, with challenges including algorithm transparency, dataset bias, integration with existing hospital IT systems, and clinician trust. These factors currently limit their widespread ICU adoption. Across all diagnostic platforms discussed, including AI-driven systems, physicians remain the central decision-makers, using diagnostic information to guide individualized patient care rather than relying on automated outputs alone.

## 4. Enhancing Antimicrobial Stewardship Through Diagnostics

Antimicrobial stewardship (AMS) refers to coordinated interventions designed to improve and measure the appropriate use of antimicrobial agents. It aims to optimize clinical outcomes while minimizing unintended consequences of antimicrobial use, such as toxicity, the selection of pathogenic organisms, and the emergence of resistance. Accurate and timely diagnostics play a pivotal role in supporting AMS, particularly in high-risk settings like ICUs, where infections are complex and time-sensitive [119].

Advanced diagnostic modalities allow clinicians to tailor antibiotic therapy to the specific pathogen and its resistance profile, reducing unnecessary broad-spectrum use and supporting evidence-based de-escalation strategies. Rapid diagnostics enable early pathogen identification, allowing for the prompt narrowing or discontinuation of empirical antibiotics. These technologies not only accelerate diagnosis but also improve clinical confidence in stewardship decisions [119,120] (Table 3).

These benefits are increasingly supported by real-world studies that demonstrate the transformative impact of diagnostic-guided stewardship in various clinical contexts. For instance, a study by Timbrook et al. found that the integration of rapid molecular diagnostics with AMS interventions significantly reduced the time to appropriate therapy and enabled faster antibiotic de-escalation [121]. Similarly, Perez et al. demonstrated that real-time AMS programs utilizing MALDI-TOF for rapid pathogen identification led to the earlier initiation of optimal antimicrobial therapy, shorter hospital stays, and reduced resource utilization [122]. Banerjee et al. also observed that combining AMS with rapid blood culture identification shortened the time to effective treatment by 20 h and was associated with lower mortality in bloodstream infections [31].

In pediatric populations, Schram et al. reported that using a rapid respiratory pathogen panel in an inpatient setting significantly reduced unnecessary antibiotic prescriptions, chest radiography, and hospital length of stay [123]. Additionally, Messacar et al. demonstrated that implementing a diagnostic AMS program for children with suspected central nervous system (CNS) infections led to substantial reductions in cerebrospinal fluid testing, antibiotic exposure, and associated costs [124]. These findings reinforce the value of diagnostics in enhancing precision care, limiting antimicrobial overuse, and reducing healthcare expenditure.

Diagnostic stewardship complements antimicrobial stewardship by ensuring that diagnostic tests are not only utilized judiciously but also interpreted correctly to guide optimal therapy. Recent evidence underscores its importance in ICU settings. For example, Bay et al. evaluated the performance of a rapid multiplex PCR panel in patients with ventilator-associated hospital-acquired pneumonia and rectal colonization with ESBL-producing Enterobacterales [125]. The implementation of this tool significantly improved the timeliness of appropriate antimicrobial therapy and reduced reliance on broad-spectrum empiric antibiotics, thereby enhancing both patient outcomes and stewardship goals [126].

Diagnostics also serve as essential stewardship metrics and are increasingly embedded into institutional clinical pathways to standardize and monitor best practices. The Centers for Disease Control and Prevention (CDC) encourages the integration of diagnostic data into stewardship dashboards to track antimicrobial usage trends, assess adherence to guidelines, and detect resistance emergence [127]. Moreover, rapid diagnostic tools have been associated with improved adherence to local protocols, including timely intravenous-to-oral antibiotic switches and appropriate empiric therapy selection [127].

This is especially critical in resource-limited settings, where diagnostic stewardship can streamline available resources, support rational empiric decision-making, and help prevent the unchecked rise of antimicrobial resistance. As healthcare systems strive to combat this global threat, integrating diagnostics into stewardship workflows is no longer optional—it is a public health imperative. Continued investment in diagnostic infrastructure, clinician education, and cross-disciplinary collaboration will be crucial to fully realize the benefits of diagnostic-driven antimicrobial management.

Real-world ICU programs demonstrate how rapid diagnostics can be integrated into stewardship workflows to produce immediate, measurable benefits. For example, implementation of the BioFire^®^ BCID panel in septic shock patients has reduced the median time to targeted therapy by over 24 h, enabling the earlier optimization or de-escalation of antibiotics and contributing to lower mortality rates [31,121].

In respiratory failure cases, rapid multiplex PCR testing of bronchoalveolar lavage (BAL) or protected telescoping catheter (PTC) samples has facilitated the narrowing of empiric broad-spectrum regimens to pathogen-directed therapy within hours. For instance, Peiffer-Smadja et al. reported that multiplex PCR for ventilator-associated and hospital-acquired pneumonia led to earlier effective antimicrobial initiation in 21% of cases and therapy de-escalation in 39% of patients [128]. The multi-center FLAGSHIP II trial by Darie et al. demonstrated that fast multiplex bacterial PCR from BAL in hospitalized pneumonia patients at risk of Gram-negative bacterial infection reduced the duration of inappropriate antibiotic therapy compared with standard culture [129]. The accompanying commentary by Dudoignon et al. further emphasized its role in enabling timely de-escalation and optimized antibiotic use in ICU pneumonia management [130].

These examples highlight that the most successful ICU stewardship models involve direct communication between the microbiology laboratory and stewardship teams, enabling results to be acted upon immediately—often during daily multidisciplinary rounds. Diagnostic-triggered protocols, such as stopping vancomycin after a negative MRSA nasal screen or initiating targeted therapy within hours of a positive resistance gene result, exemplify how rapid test data can be translated into prompt and impactful clinical decisions.

## 5. Barriers and Limitations

While diagnostic innovations have significantly advanced the potential for targeted antimicrobial therapy, several barriers limit their full integration into routine clinical practice.

A major barrier is the high cost associated with implementing and maintaining these advanced diagnostic technologies. Many healthcare facilities, particularly in low- and middle-income countries, lack the financial resources to acquire the necessary platforms or sustain their use over time. Even in high-income settings, the upfront capital investment for equipment, software integration, and staff training can be difficult to justify without clear immediate returns. Moreover, ongoing costs such as reagents, calibration, and quality control can strain hospital budgets, especially where reimbursement systems do not adequately account for diagnostic stewardship benefits [119,124].

Infrastructural and human resource limitations further impede the effective deployment of diagnostic innovations. Successful implementation requires not only the technology itself but also a supportive ecosystem that includes reliable laboratory facilities, trained personnel, and rapid communication pathways between the laboratory and clinical teams. In many hospitals, particularly those with limited microbiology support or 24/7 lab coverage, the turnaround time and interpretation of diagnostic results may still delay appropriate therapy decisions. A lack of trained infectious disease specialists and clinical microbiologists can also result in the underutilization or misinterpretation of test results, weakening the impact of these tools on patient care [131,132].

Another critical challenge lies in the complexity of interpreting diagnostic findings in the clinical context. Many rapid diagnostic tools detect genetic material or specific microbial markers without necessarily indicating active infection. For example, molecular assays for *Clostridioides difficile* or respiratory viruses may detect colonization rather than active disease, which can lead to overdiagnosis and unnecessary treatment if not interpreted cautiously. This limitation underscores the need for integrated diagnostic algorithms that combine laboratory findings with clinical presentation to guide therapy decisions effectively [133].

Regulatory and logistical disparities also pose significant obstacles, particularly in low- and middle-income countries. Many of the most effective and innovative diagnostic tools are developed and validated in high-income countries, with little adaptation or access pathways available for resource-constrained settings. Regulatory bottlenecks, the absence of local manufacturing, and reliance on imported diagnostics further delay deployment, increasing the risk of antimicrobial misuse in these regions where empiric broad-spectrum therapy remains the norm [119].

Finally, behavioral and organizational barriers play a crucial role in limiting the adoption of diagnostic-guided therapy. Clinicians may be hesitant to rely on new diagnostic tools if they lack confidence in their accuracy, do not fully understand their clinical utility, or are accustomed to traditional empiric treatment strategies. In addition, institutional inertia, insufficient leadership support, and fragmented communication between laboratory and clinical departments can result in the slow uptake or inconsistent application of diagnostics in decision-making. Promoting a culture of diagnostic stewardship requires ongoing education, interdisciplinary collaboration, and the inclusion of diagnostics into clinical guidelines and stewardship metrics [134,135,136,137].

In summary, although diagnostic innovations offer transformative potential for enabling timely, targeted antimicrobial therapy, their impact is curtailed by financial, infrastructural, interpretative, regulatory, and behavioral challenges. Addressing these barriers through investment in diagnostic infrastructure, clinician training, regulatory streamlining, and integration of diagnostics into stewardship frameworks is essential to fully realize their value in the fight against antimicrobial resistance.

## 6. Conclusions and Future Perspectives

In the face of escalating antimicrobial resistance, diagnostic innovations are no longer secondary tools but essential pillars of infection management in critical care. The transition from empirical to evidence-based therapy relies heavily on the ability to rapidly identify pathogens and resistance mechanisms, a goal now increasingly attainable through technologies like multiplex PCR, MALDI-TOF MS, metagenomics, and point-of-care diagnostics. These tools not only expedite clinical decision-making but also serve as integral components of AMS frameworks, helping to preserve the efficacy of current antibiotics and improve patient outcomes.

However, realizing the full potential of these diagnostic advancements requires overcoming significant implementation barriers. High costs, infrastructure limitations, diagnostic interpretation challenges, and disparities in global access remain persistent obstacles. Furthermore, integrating these tools into routine ICU practice necessitates ongoing clinician education, multidisciplinary collaboration, and alignment with institutional stewardship goals. Addressing these limitations is necessary to optimize diagnostic utility in ICU settings.

Looking ahead, future advancements must prioritize affordability, scalability, and interoperability. AI and ML are increasingly being explored for their potential to refine prediction models, support individualized therapy, and inform surveillance strategies; however, most applications remain in the early stages of validation and require further evidence before routine ICU adoption. Strengthening the integration of validated diagnostic tools into stewardship frameworks represents a more immediate priority for optimizing antimicrobial use and patient outcomes.

## Figures and Tables

**Table 1:** Clinical readiness of emerging diagnostic tools in critical care.

Technology	Primary Function	Readiness Level	Notes on Adoption/Limitations
BioFire^®^ BCID panel	Pathogen + resistance gene detection	Established	Widely implemented in ICUs; rapid bloodstream infection diagnosis; higher cost offset by improved outcomes
Cepheid GeneXpert^®^	Pathogen + resistance gene detection	Established	Routine ICU screening for MRSA, carbapenemase producers; rapid turnaround
Lateral flow assays (LFAs)	Pathogen detection	Established	Common for *C. difficile*, *Legionella*, *S. pneumoniae*, influenza, SARS-CoV-2; lower sensitivity than molecular tests
Multiplex LFAs with digital readers	Pathogen detection	Emerging	Undergoing multi-center trials; not yet standard ICU tools
Biosensors for pathogen ID	Pathogen detection	Experimental	Prototype stage; limited clinical validation
LOC platforms for AST	Resistance detection	Emerging	Early pilots; potential for rapid susceptibility testing
Phenotypic biosensors for resistance	Resistance detection	Emerging	Limited ICU adoption; promising in validation studies
Nucleic acid-based biosensors	Resistance detection	Experimental	Early-stage testing; limited integration
AI-powered AMR predictive tools	Resistance prediction and surveillance	Experimental	Research and pilot settings; not standardized
Smartphone-integrated diagnostics	Pathogen/resistance detection	Experimental	Mostly in research or limited pilot deployment
Procalcitonin (PCT), CRP assays	Host biomarker detection	Established	Widely used for guiding antibiotic therapy duration
Biosensor-based host biomarker platforms	Host biomarker detection	Emerging	Undergoing validation for ICU point-of-care use

**Table 2:** Comparative overview of diagnostic technologies for critical care settings.

Technology	Primary Function	Typical Turnaround Time	Accuracy/Sensitivity	Cost Category *	Readiness Level	Potential Clinical Impact
Lateral flow assays (LFAs)	Rapid pathogen detection (antigens/toxins)	10–30 min	Moderate (60–85%)	Low	Established	Enables early targeted therapy; reduces unnecessary antibiotics in high-prevalence settings
Multiplex LFAs (enhanced sensitivity)	Simultaneous detection of multiple pathogens	20–45 min	Moderate–High (75–95%)	Moderate	Emerging	Improves diagnostic yield for complex infections; still in validation
Biosensors for pathogen ID	Direct detection of microbial antigens, VOCs, or nucleic acids	15–60 min	High (>90%) in controlled settings	Moderate–High	Experimental	Potential for bedside, real-time diagnosis without lab infrastructure
Lab-on-a-chip (LOC) for AST	Rapid phenotypic antimicrobial susceptibility testing	1–4 h	High (>90%)	High	Emerging	Reduces time to targeted therapy; may improve sepsis outcomes
Molecular panels (BioFire^®^, GeneXpert^®^)	Syndromic pathogen + resistance gene detection	~1 h	Very high (>95%)	High	Established	Speeds targeted therapy; shortens ICU stays; supports infection control
Phenotypic biosensors for resistance	Detects enzymatic resistance (e.g., carbapenemase activity)	<1 h	High (>90%)	Moderate	Emerging	Facilitates rapid stewardship decisions for MDR infections
Nucleic acid-based biosensors	Detects genetic resistance markers	30–90 min	High (>90%)	Moderate–High	Experimental	Enables point-of-care genotypic resistance detection
Smartphone-based diagnostics	Portable detection + digital integration	15–60 min	Moderate–High	Low–Moderate	Experimental	Promising for remote/low-resource ICUs; enables real-time data sharing
Procalcitonin (PCT), CRP assays	Host biomarker detection for infection severity	<1 h	High (>90%)	Moderate	Established	Guides antibiotic initiation/de-escalation; reduces overuse
Biosensor-based host biomarker platforms	Rapid bedside biomarker detection	15–45 min	High (>90%)	Moderate–High	Emerging	Potential for real-time severity monitoring in ICU
AI-powered predictive tools	AMR risk prediction and therapy optimization	Instant–few min (data-dependent)	Variable (depends on model)	Moderate	Experimental	Supports precision prescribing; enhances stewardship surveillance

* Cost Category is indicative and may vary by setting and procurement agreements.

**Table 3:** Impact of diagnostic stewardship interventions on clinical and antimicrobial outcomes. ↓ "decrease", ↑ "increase".

Study	Setting	Diagnostic Tool	Key Outcomes
Timbrook et al. [121]	ICU	Rapid molecular diagnostics + AMS	↓ Time to therapy, ↑ antibiotic de-escalation
Perez et al. [122]	General hospital	MALDI-TOF + real-time AMS	↓ Time to optimal therapy, ↓ hospital length of stay (LOS), ↓ resource use
Banerjee et al. [31]	Bloodstream infections	Rapid blood culture ID + AMS	↓ Time to effective therapy (by 20 h), ↓ mortality
Schram et al. [123]	Pediatric inpatient	Rapid respiratory panel	↓ Unnecessary antibiotics, ↓ imaging, ↓ LOS
Messacar et al. [124]	Pediatric CNS infection	Diagnostic AMS program	↓ CSF testing, ↓ antibiotic exposure, ↓ hospital costs
Bay et al. [125]	ICU, ventilator-associated pneumonia	Rapid multiplex PCR (ESBL carriers)	↑ Timely therapy, ↓ empiric broad-spectrum antibiotic use
